# Anti-Glycation Activities of *Angelica keiskei* Leaves

**DOI:** 10.3390/molecules30061394

**Published:** 2025-03-20

**Authors:** Yuno Takemoto, Takashi Kikuchi, Wenjun Qi, Mi Zhang, Kouharu Otsuki, Wei Li

**Affiliations:** Faculty of Pharmaceutical Sciences, Toho University, Miyama 2-2-1, Funabashi 274-8510, Chiba, Japan

**Keywords:** LC-MS, *Angelica keiskei*, anti-glycation activity, advanced glycation end products

## Abstract

The screening of a small library of Japanese herbal tea extracts revealed significant anti-glycation activity in the leaves of *Angelica keiskei*. LC-MS analysis led to the identification of twenty compounds in this herb tea, including seven flavonoids, five phenylpropanoids, and eight coumarin derivatives, based on their chromatographic behavior and fragmentation patterns. Further LC-MS analysis of the methanol-eluted fraction after incubation with methylglyoxal (MGO) was performed on the reaction mixture, revealing quercetin 3-*O*-glucoside to be a key compound contributing to the anti-glycation activity of the leaves.

## 1. Introduction

Advanced glycation end products (AGEs) are formed through a non-enzymatic reaction, known as glycation, between reducing sugars and proteins. The reaction between reducing sugars and the free amino groups of proteins leads to the formation of Schiff bases, followed by the production of Amadori products and ketoamines [1,2,3,4]. Additionally, oxidative, dehydration, and other cross-linking reactions produce reactive dicarbonyl compounds. These dicarbonyl compounds exhibit high reactivity with amino acids such as arginine and lysine in proteins, contributing to the formation of AGEs [3].

Reactive carbonyl species, such as glyoxal, methylglyoxal (MGO), and 3-deoxyglucosone, are generated through various metabolic pathways in the human body. Among them, MGO is a highly reactive α-dicarbonyl compound endogenously produced during glycolysis [5]. Hyperglycemia in diabetic and obese patients significantly increases the MGO levels in plasma and urine as a result of glycolytic overload [6]. The abnormal accumulation of MGO, referred to as dicarbonyl stress, is potentially associated with various diseases [7]. MGO promotes post-translational modifications of peptides and proteins, ultimately leading to the formation of AGEs. Eliminating reactive dicarbonyl compounds is considered an effective strategy for preventing protein modification and AGE formation. Synthetic inhibitors, such as aminoguanidine and metformin, exhibit potent anti-glycation activity but raise concerns about the potential side effects. In contrast, herbs and natural compounds derived from them tend to have relatively fewer side effects as inhibitors of AGE formation [8].

*Angelica keiskei* Koidz. is a perennial herb native to Japan, thriving in the country’s warm coastal regions. The plant grows to a height of approximately 80–120 cm, releasing a yellow sap when its stems or leaves are cut. The young leaves of the *A. keiskei* in early spring are edible, and the dried leaves are used as a herbal tea. The leaves have been used in folk medicine for their diuretic, mild laxative, and capillary-strengthening effects [9]. The aerial parts of *A. keiskei* have been reported to contain chemical constituents, including flavonoids, coumarins, phenolic compounds, acetylenes, sesquiterpenes, and triterpenes, along with various biological activities such as anti-inflammatory, anti-obesity, anti-hyperlipidemic, anti-oxidative, anti-thrombotic, anti-tumor and anti-mutagenic, anti-bacterial, and hepatoprotective effects [10]. In the leaves, chalcones and coumarin derivatives have also been reported [11,12]. Pharmacological investigations have reported that *A. keiskei* exhibited anti-diabetic activity as follows: the ethanol extract of the leaves has been shown to significantly lower fasting blood glucose levels and markedly improve glucose tolerance in diabetic mice [13]; the ethanol extract of the roots has been reported to exhibit insulin-like activities via a pathway independent of peroxisome proliferator-activated receptor-γ activation [14]; chalcones from *A. keiskei* have been reported to inhibit PTP1B [15] and improve insulin resistance in type 2 diabetic rats [16].

During the screening of a small library of Japanese herbal tea extracts, *A. keiskei* leaf tea exhibited anti-glycation activity. Therefore, this study aimed to elucidate the anti-glycation activity of *A. keiskei* leaves. The inhibitory activity of *A. keiskei* leaves on AGE production was evaluated using an MGO-trapping assay. The chemical constituents were then identified via LC-MS analysis. Furthermore, a comparative analysis of the MeOH-eluted fraction treated with and without MGO was conducted using LC-MS, leading to the identification of the key compounds responsible for the anti-glycation activity.

## 2. Results and Discussions

### 2.1. Inhibitory Activity of AGE Production by A. keiskei Leaves

**Figure 1:**
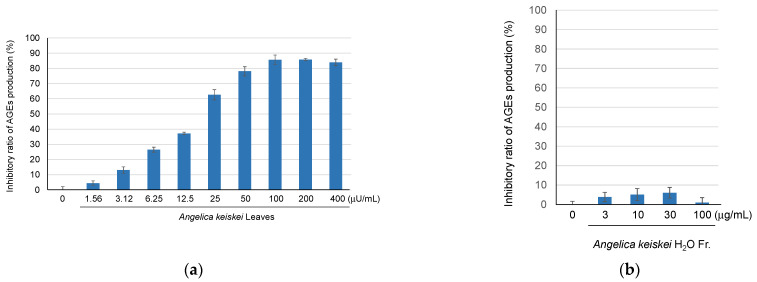

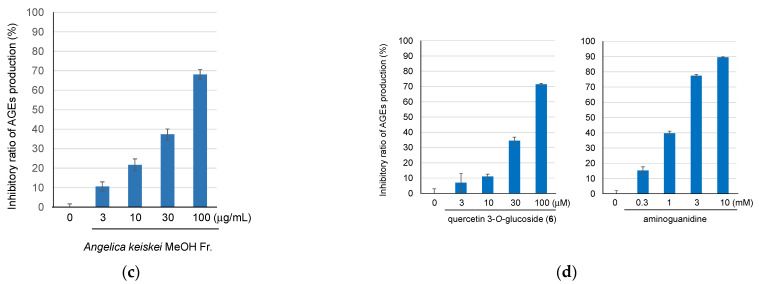
The inhibitory ratio of AGE production of *A. keiskei* leaves extract (**a**), and its H_2_O (**b**), MeOH-eluted fractions (**c**), quercetin 3-*O*-glucoside (**6**), and the positive control, aminoguanidine (**d**).

The inhibitory activity of *A. keiskei* leaves on AGE production has been reported in several assays, including glucose-BSA, fructose-BSA, and glucose-arginine assays [17]. In this study, the inhibitory activity was evaluated using an MGO-trapping assay. The hot water extract of *A. keiskei* leaves exhibited anti-AGE production activity in a concentration-dependent manner (Figure 1a). Furthermore, two fractions, the H_2_O-eluted fraction and the MeOH-eluted fraction, were obtained from the extract using Diaion HP-20 column chromatography. Upon evaluating the anti-glycation activity, the water-eluted fraction showed no activity (Figure 1b), whereas the MeOH-eluted fraction exhibited concentration-dependent anti-glycation activity (Figure 1c). These results suggested that the anti-glycation compounds of *A. keiskei* leaves were present in the MeOH-eluted fraction.

### 2.2. Identification of Compounds in A. keiskei Leaves Using LC-MS and LC-MS/MS Analyses

**Figure 2:**
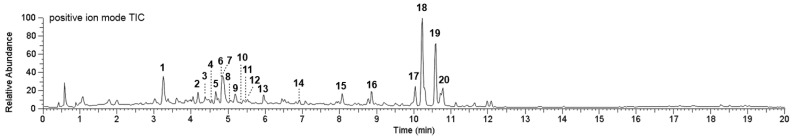
LC-MS analysis of the MeOH-eluted fraction from *A. keiskei* leaves.

**Figure 3:**
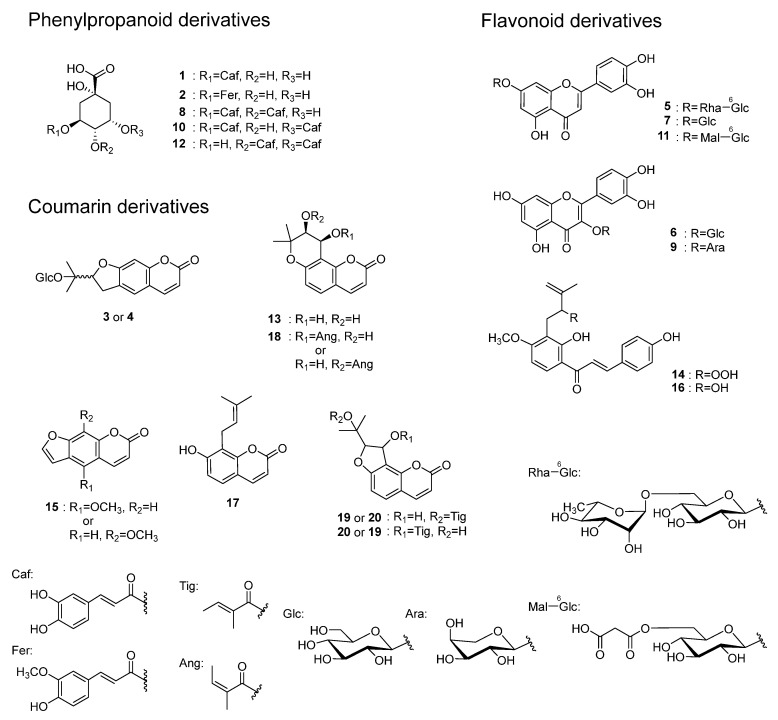
Structures of compounds identified in *A. keiskei* leaves.

The MeOH-eluted fraction obtained from the hot water extract of *A. keiskei* leaves, which exhibited anti-AGE production activity, was analyzed via LC-MS to identify the chemical constituents. As a result, 20 compounds, including five phenylpropanoid derivatives (**1**, **2**, **8**, **10**, **12**), eight coumarin derivatives (**3**, **4**, **13**, **15**, **17**, **18**, **19**, **20**), and seven flavonoid derivatives (**5**, **6**, **7**, **9**, **11**, **14**, **16**) (Figure 2 and Figure 3) were identified via the detailed analysis of their chromatographic behavior and fragmentation patterns (Table 1).

**Figure 4:**
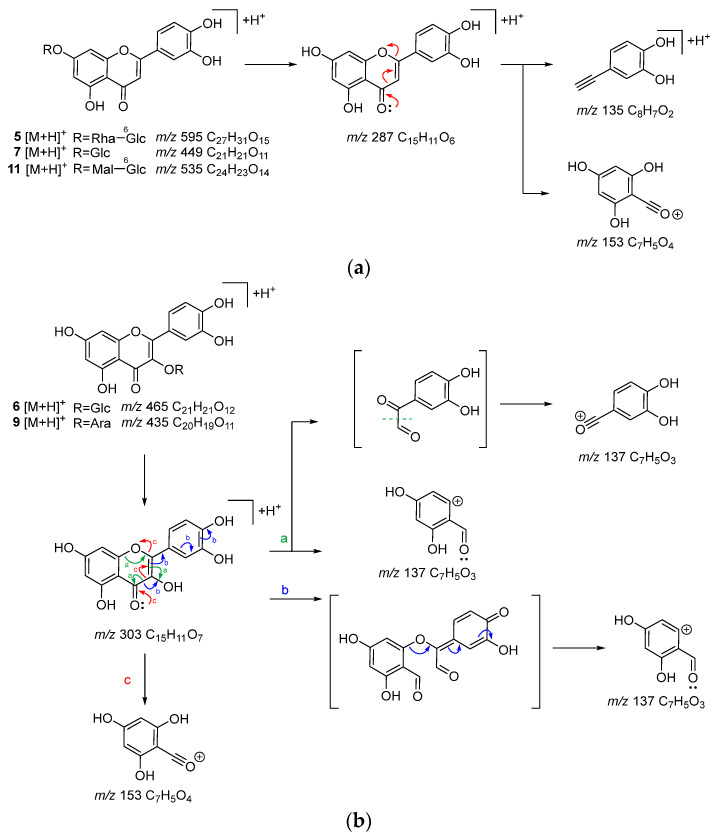
Possible fragmentation pathways of flavonoids **5**, **7**, and **11** (**a**) and **6** and **9** (**b**) in *A. keiskei* leaves (positive ion mode).

Compounds **5**, **6**, **7**, **9**, **11**, **14**, and **16** are flavonoids. In the MS/MS analysis using [M + H]^+^ as the precursor ion, compounds **5**, **7**, and **11** showed product ions derived from the A-ring and B-ring (*m*/*z* 153, 135) following the loss of the sugar moiety and the subsequent cleavage of the C-ring (Figure 4a). Based on this, the aglycone of these compounds was identified as luteolin. For compound **5**, the [M + H]^+^ MS/MS spectrum at HCD 10 eV showed an *m*/*z* 449 ion (C_21_H_21_O_11_), indicating the loss of a rhamnose moiety, suggesting that it possesses a rhamnosyl-glucose sugar chain structure. In the MS of compound **11**, the fragment ion corresponding to the loss of malonic acid was also observed. For compounds **6** and **9**, product ions corresponding to the protonated aglycone, quercetin (*m*/*z* 287), as well as product ions derived from the A-ring (*m*/*z* 153) and B-ring (*m*/*z* 137) resulting from C-ring cleavage, were also observed (Figure 4b). The neutral loss suggested that the detached sugar moieties were a glucose moiety for compounds **6** and **7**, and arabinose moiety for compound **9**. In the MS/MS of [M + H]^+^ from compound **16**, product ions were observed as a result of the α-cleavage of the carbonyl group, leading to ions being derived from the A-ring (*m*/*z* 235), as well as ions corresponding to the C_6_C_3_ moiety including the B-ring (*m*/*z* 147). Additionally, product ions corresponding to the loss of CO and H_2_O were detected (Figure S5). For compound **14**, product ions were observed corresponding to the loss of H_2_O from the hydroperoxy group, α-cleavage of the carbonyl group (*m*/*z* 233, 147), and the loss of CO, a methyl group, and an isopropenyl group (*m*/*z* 179, 119). Furthermore, ions derived from the loss of a methyl group, α-cleavage of the carbonyl group producing A-ring-derived ions, and the loss of OOH from the [M + H]^+^ ion were also observed (*m*/*z* 195, 163) (Figure S4). Compounds **14** and **16** are chalcones unique to *A. keiskei* [15]. Compounds **5**, **6**, **7**, and **9** were isolated from *A. keiskei* [18], while compound **11** is unreported from the *Angelica* genus.

Compounds **3**, **4**, **13**, **18**, **15**, **17**, **19**, and **20** are coumarin derivatives. In the MS/MS analysis of coumarin derivatives, compounds **3** and **4** showed product ions resulting from the sequential loss of a glucose moiety, H_2_O, and a methyl group (*m*/*z* 299, 213) when using [M + H]^+^ as the precursor ion. Compounds **3** and **4** have been isolated from the *Angelica* genus [19]. For compounds **19** and **20**, MS/MS using [M + H]^+^ as the precursor ion revealed ions corresponding to the loss of a tigloyl group and an isopropenyl group (*m*/*z* 229, 187). Product ions derived from the tiglic acid part and resulting from the subsequent loss of CO (*m*/*z* 83, 55) were also observed (Figure S7). In compounds **13** and **18**, MS/MS using [M + H]^+^ as the precursor ion showed ions resulting from the loss of a C-CO, (CH_3_)_2_C-CO moieties, and H_2_O (*m*/*z* 203, 175). Additionally, in compound **18**, product ions corresponding to the loss of an angelic acid moiety were also observed (Figure S3). In compound **17**, product ions were observed resulting from the sequential loss of the (CH_3_)_2_=C moiety and CO (*m*/*z* 175, 147, 119, 91) (Figure S6), which matched those reported previously [20]. Compound **15** was identified as bergapten or methoxsalen from its molecular formula and MS/MS fragmentation [21], a known compound of *A. keiskei*. Compounds **13**, **15**, **17**, **18**, **19**, and **20** were isolated from *A. keiskei* [15,22,23,24].

Compounds **1**, **2**, **8**, **10**, and **12** are phenylpropanoid derivatives. Based on the molecular formula obtained from high-resolution ESI-MS, compound **1** was presumed to be chlorogenic acid (**1**: C_16_H_26_O_9_), which has been reported as the compound included in *A. keiskei* [25]. In the MS/MS analysis using [M + H]^+^ as the precursor ion for compound **1**, product ions resulting from the sequential loss of a caffeic acid (*m*/*z* 163), CO, and OH moieties were observed (Figure S1), which matched the standard compound. For compounds **8** and **12**, similar product ions to those of compound **1** were detected. Based on their molecular formulas and their chromatographic behavior [26], compounds **8**, **10**, and **12** were estimated to be 3,4-dicaffeoylquinic acid, 3,5-dicaffeoylquinic acid, and 4,5-dicaffeoylquinic acid (C_25_H_24_O_5_). In the case of compound **2**, product ions corresponding to the loss of a feruric acid moiety (*m*/*z* 177) were observed, leading to its identification as feruloylquinic acid (C_17_H_20_O_9_) (Figure S1). Compounds **2**, **8**, and **12** have been reported to occur in the *Angelica* genus [21,27,28]

Of the identified compounds, it was the first time for **11** to be identified in the *Angelica* genus, while it was the first time for **2**, **3**, **4**, **8**, and **12** to be identified in *A. keiskei*.

### 2.3. Identification of the Key Compounds in A. keiskei Leaves for the Inhibitory Activity of AGE Production

**Figure 5:**
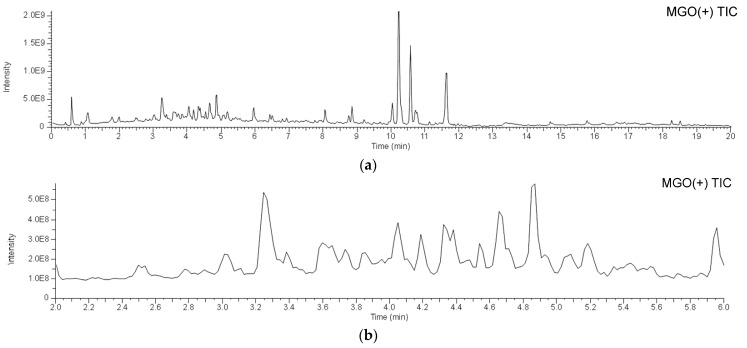

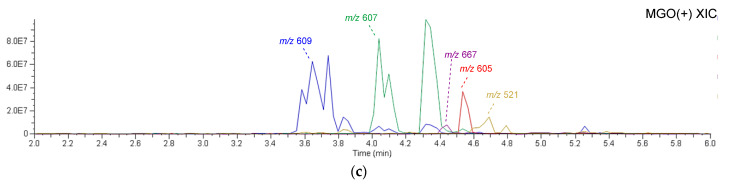
LC-MS of *A. keiskei* leaves extract after incubation with methylglyoxal. (**a**) 0–20 min; (**b**) 2.0–6.0 min; (**c**) extracted ion chromatogram (XIC) of *m*/*z* 609, 607, 667, 605, and 521 in 2.0–6.0 min.

**Figure 6:**
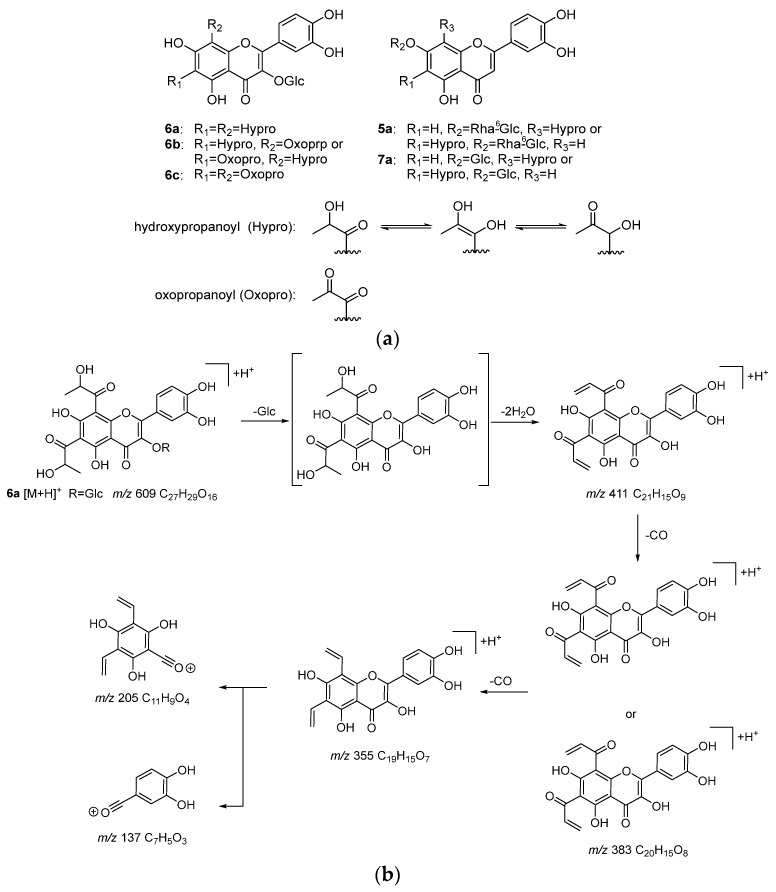
Products (**5a**, **6a**–**6c**, and **7a**) from incubation of MeOH-eluted fraction of *A. keiskei* with MGO (**a**), and MS/MS fragmentation pathway of the reaction product **6a** (**b**).

To identify the bioactive compounds contributing to the antiglycation activity of *A. keiskei* leaves, the total chromatograms of the MeOH-eluted fraction from *A. keiskei* leaves with or without MGO were compared to observe a decrease in the peak intensity of MGO-scavenging compounds and the appearance of a corresponding MGO-binding compound peak upon the addition of MGO. The addition of MGO causes a decrease in the peak intensity of the MGO-scavenging compound and the appearance of the corresponding MGO-bound compound peak. Upon the addition of MGO, the peak intensity of compound **6** decreased, while peaks presumed to be the products of MGO trapping were observed around 3.4–4.9 min (Figure 5a,b). The main product peaks were *m*/*z* 609 (**6a**), *m*/*z* 607 (**6b**), and *m*/*z* 605 (**6C**) (Figure 5c). These peaks are considered to correspond to products in which compound **6** trapped two MGO molecules. The hydroxypropanoyl moiety of **6a** and **6b** is thought to exist in equilibrium structures (Figure 6a).

In the MS/MS analysis of **6a** using [M + H]^+^ as the precursor ion, product ions resulting from the sequential losses of the glucose moiety, H_2_O, and CO (*m*/*z* 411, 383, 355) were observed (Figure 6b). Additionally, product ions generated via C-ring cleavage (*m*/*z* 205, 137) were detected. The ion at *m*/*z* 137, which was also observed in the MS/MS analysis of compound **6**, was observed and was interpreted as originating from the B-ring. On the other hand, the ion at *m*/*z* 205, based on its molecular formula (C_11_H_9_O_4_), was interpreted as having two vinyl groups attached to the ion at *m*/*z* 153 (C_7_H_5_O_4_) observed in the MS/MS of compound **6**. These vinyl groups are considered to result from the elimination of CO and H_2_O from the hydroxypanoyl group bonded via the reaction with MGO. This suggests that one molecule of **6** trapped two molecules of MGO at the A-ring.

In **6b** and **6c**, the product ions derived from the loss of the glucose moiety, oxopropanoyl group, and H_2_O, as well as CO in the case of **6b**, were observed (**6b**: *m*/*z* 357, 329; **6c**: *m*/*z* 373, 355). Additionally, the ions at *m*/*z* 179 in **6b** and *m*/*z* **205** in **6c** supported the notion that MGO was also trapped at the A-ring of **6b** and **6c**. Conversely, the ion at *m*/*z* 137, interpreted as originating from the B-ring, was also observed in **6b** and **6c**, indicating that the B-ring did not trap MGO.

Furthermore, small peaks were observed around 4.45 min and 4.70 min, which were identified as **5a** (*m*/*z* 667: C_30_H_35_O_17_) and **7a** (*m*/*z* 521: C_24_H_25_O_13_), respectively (Figure 5c and Figure 6a). Based on their molecular formulas, these were presumed to be products formed via the reaction of one molecule of MGO with compounds **5** and **7**, respectively. In the MS/MS analysis using [M + H]^+^ as the precursor ion, product ions such as *m*/*z* 135, interpreted as originating from the B-ring, which was observed in the MS/MS of compounds **5** and **7**, were detected. Additionally, *m*/*z* 179, interpreted as originating from the A-ring and indicating the binding of vinyl groups to the ion at *m*/*z* 153, was observed. These findings suggest that MGO was also bound to the A-ring of compounds **5** and **7**. However, their ion intensities were weaker than those of the products derived from compound **6**.

The MGO-trapping activity of flavonoids depends on the nucleophilic reaction between flavonoids and MGO. Regarding the MGO-trapping ability of flavonoids, structure–activity relationships have been discussed as follows: (i) the A-ring contributes to MGO trapping, and the hydroxyl group at the C-5 position enhances the trapping activity; (ii) the double bond between C-2 and C-3 in the C-ring may enhance the MGO trapping; (iii) the number of hydroxyl groups in the B-ring has little influence on the MGO trapping [29]. In this study, compounds **5**–**7**, which trapped MGO, all possess a double bond between C-2 and C-3 as well as a hydroxyl group at the C-5 position, supporting the previous findings. In addition, while compound **6**, which has a sugar moiety at the 3-OH position, trapped two molecules of MGO in one molecule, compounds **5** (*m*/*z* 667) and **7** (*m*/*z* 521), which have sugar chains at the 7-OH position, trapped only one molecule of MGO each.

Compound **6**, which exhibited a decrease in the peak intensity upon the addition of MGO, was evaluated for its AGE formation inhibitory activity. It was found to have a stronger inhibitory activity (IC_50_ 49.2 µM) than the positive control aminoguanidine (IC_50_ 1.52 mM) (Figure 1d). Flavonoids, including compound **6,** showed a relatively high intensity in Figure 2, suggesting that the activity potency and content of these flavonoid glycosides contribute to the anti-glycation activity of *A. keiskei* leaves. One of the mechanisms underlying the AGE formation inhibitory activity of *A. keiskei* leaves involves the carbonyl-trapping ability of flavonoid glycosides such as **6**.

## 3. Materials and Methods

### 3.1. General Methods

Fluorescence intensity was measured using an Infinite 200Pro M Nano+ [Tecan Japan Co., Ltd. (Kanagawa, Japan)]. LC-MS analysis was performed on a Vanquish UHPLC system combined with a Q-Exactive Hybrid Quadrupole Orbitrap mass spectrometer (Thermo Scientific, Waltham, MA, USA). Column chromatography was performed on Diaion HP-20 (Mitsubishi Chemical Corporation, Tokyo, Japan). Methylglyoxal was purchased from Nacalai Tesque, Inc. (Kyoto, Japan). FUJIFILM Wako Pure Chemical Corporation (Osaka, Japan) supplied 0.1 moL/L phosphate buffer, acetic acid, human serum albumin, and dimethyl sulfoxide. Sodium azide and aminoguanidine were purchased from Sigma-Aldrich Japan Co. (Tokyo, Japan). Chlorogenic acid hydrate (>98%) was purchased from Tokyo Chemical Industry Co., Ltd. (Tokyo, Japan) and quercetin 3-*O*-glucoside (99.71%) was purchased from Selleck Chemicals (Houston, TX, USA). LC-MS grade acetonitrile, methanol, and distilled water were purchased from Kanto Chemical Co., Inc. (Tokyo, Japan).

### 3.2. Materials

*A. keiskei* herbal tea leaves, produced in Tokushima prefecture in Japan, were purchased from Kawamotoya Shoten (Kanagawa, Japan) in 2021.

### 3.3. Preparation of the Sample Solutions

The extract obtained via the hot water extraction of 20 g of health tea in 1 L for 45 min and then added to 1 L of water was defined as 1 U/L. This concentration is approximately equivalent to that of tea when consumed as a beverage. To evaluate the anti-glycation activity, a 1 U/L water extract of *A. keiskei* tea was prepared using 10 g of *A. keiskei* tea and 500 mL of water.

### 3.4. Assay for the Anti-Glycation Activity Using Methylglyoxal

The evaluation method was partially modified based on a previously reported method [30]. HSA was used as the protein, MGO, an intermediate of sugar metabolism, as the sugar, and aminoguanidine (AG), as the positive control. These and sample solutions were prepared at each concentration with 50 mM phosphate buffer (pH 7.4) containing 0.02% NaN_3_. An HSA solution (125 µL, final concentration (FC) 1.0 mg/mL) and sample solution (100 µL) were added to each well. An MGO solution (25 µL, FC 0.04 mg/mL) or buffer (25 µL) was then added to the wells, and the plate was incubated at 37 °C for 24 h. The fluorescence intensity was measured at an excitation wavelength of 370 nm and an emission wavelength of 440 nm. The inhibition rate of AGE formation (%) was calculated using the following equation:

AGEs inhibition ratio (%) = [1 − (Abs sample with MGO − Abs sample without MGO)/Abs vehicle] × 100.

### 3.5. Fractionation of A. keiskei Leaves Extract

The extract (1 U/L, 5 mL) was subjected to a Diaion HP-20 column chromatography eluted with H_2_O and MeOH, yielding H_2_O elute (6.5 mg) and MeOH eluate fractions (2.0 mg).

### 3.6. LC-MS Analysis

LC-MS analysis was performed using a Vanquish UHPLC system equipped with a Q-Exactive hybrid quadrupole orbitrap high-resolution accurate mass spectrometer.

In the LC section, the flow rate was set at 0.4 mL/min, and the column temperature was maintained at 40 °C. Chromatographic separation was carried out using a TSKgel ODS-120H column (100 × 2.0 mm I.D., 1.9 μm, Tosoh Corporation, Tokyo, Japan). The mobile phase consisted of solvent A (distilled water with 0.1% formic acid) and solvent B (acetonitrile with 0.1% formic acid), with a gradient elution program as follows: 0–18 min, 5%→100% B; 18–20 min, 100% B. The injection volume was 2 μL.

In the MS section, measurements were conducted in both positive and negative ion modes using ESI. The calibration of the ESI-MS was performed with calibration solutions. The mass spectrometry parameters were set as follows: spray voltage, +3.5 kV (positive ion mode) and −2.5 kV (negative ion mode); capillary temperature, 262.5 °C; sheath gas flow rate, 50 units; AUX gas flow rate, 12.5 units; sweep gas flow rate, 2.63 units; S-lens RF level, 50 units; and probe heater temperature, 425 °C. The in-source CID was set at 0 eV.

Data acquisition was conducted in the Full MS mode and Full MS/data-dependent (dd) MS/MS mode. The resolution was 70,000 for Full MS and 35,000 for Full MS/dd-MS/MS. AGC was configured to 1 × 10^6^ for Full MS and 1 × 10^5^ for dd-MS/MS. The maximum ion injection time was set to 200 ms for Full MS. The scan range for Full MS was set to 150–2000 *m*/*z*. The dd-MS/MS scans were performed using high-energy collisions (HCD) with normalized collision energies (NCE) of 10, 40, and 60 eV.

### 3.7. Evaluation of the MGO Trapping Capacity of A. Keislei Leaves

*A. keiskei* leaves (10 g) were extracted according to the method described in Section 3.3, followed by Diaion HP-20 column chromatography as described in Section 3.4, yielding the MeOH eluate fraction (235.5 mg). The *A. keiskei* leaves MeOH elute fraction (10 mg) was incubated with MGO (150 mM or 0 mM) in PBS buffer (pH 7.4, 50 mM) at 37 °C. After 24 h of incubation, it was subjected to Diaion HP-20 column chromatography to give the MeOH-eluted part. The MeOH-eluted part was evaporated in vacuo to yield a MeOH-eluted fraction (10.6 mg). The MeOH-eluted fraction in methanol (1 mg/mL) was filtrated with a 0.22 μm filter and then analyzed using LC-MS.

## 4. Conclusions

This study revealed that the hot water extract of *A. keiskei* leaves exhibited anti-glycation activity. LC-MS analysis identified twenty compounds in the MeOH-eluted fraction, including five phenylpropanoid derivatives, eight coumarin derivatives, and seven flavonoid derivatives. Furthermore, in the MeOH-eluted fraction of *A. keiskei* leaves treated with MGO, MGO-adducts derived from quercetin 3-*O*-glucoside (**6**), a major compound of *A. keiskei* leaves, as well as luteolin 7-*O*-rutinoside (**5**) and luteolin 7-*O*-rutinoside (**7**), were detected, suggesting their contribution to the activity of *A. keiskei* leaves. Additionally, compound **6** exhibited anti-glycation activity. These results suggest that carbonyl trapping by flavonoid glycosides may be one of the mechanisms underlying the anti-glycation effects of *A. keiskei* leaves. Regarding the carbonyl trapping of flavonoid glycosides in vivo, it has been reported that after the oral intake of rutin, a glycoside of quercetin, rutin-MGO adducts reached their maximum plasma concentration 15 min after ingestion and then gradually decreased over time [31]. In another study, the oral administration of the total flavonoids from the dry leaves of *Apocynum venetum* L., which contain high levels of flavonoid glycosides, mainly quercetin glycosides and kaempferol glycosides, to C57BL/6J mice resulted in the detection of MGO adducts of the deglycosylated forms and their derivatives in fecal samples [32]. Flavonoid glycosides in *A. keiskei* can also be expected to trap MGO through a similar metabolic process. To fully elucidate the health-promoting potential of *A. keiskei* leaves, further research is needed to investigate the kinetics of digestion, the bioavailability, and bioaccessibility of its compounds and metabolites in vivo.

## Figures and Tables

**Table 1:** Compounds identified from *A. keiskei* via LC-MS.

		Molecular	ESI-MS (+)		ESI-MS (−)		Identification
No.	t_R_ (min)	Formula	*m*/*z*	Adduct Ion	Mass (*m*/*z*)	Adduct Ion	
**1**	3.25	C_16_H_18_O_9_	355.1020	[M + H]^+^	353.0880	[M − H]^−^	chlorogenic acid ^c^
**2**	4.19	C_17_H_20_O_9_	369.1177	[M + H]^+^	367.1035	[M − H]^−^	feruloylquinic acid
**3**	4.38	C_20_H_24_O_9_	409.1490	[M + H]^+^	453.1407	[M + HCOO]^−^	Nodakenin ^a^
**4**	4.56	C_20_H_24_O_9_	409.1490	[M + H]^+^	453.1407	[M + HCOO]^−^	Marmesinin ^a^
**5**	4.67	C_27_H_30_O_15_	595.1655	[M + H]^+^	593.1516	[M − H]^−^	luteolin 7-*O*-rutinoside
**6**	4.82	C_21_H_20_O_12_	465.1028	[M + H]^+^	463.0886	[M − H]^−^	quercetin 3-*O*-glucoside ^c^
**7**	4.86	C_21_H_20_O_11_	449.1070	[M + H]^+^	447.0935	[M − H]^−^	luteolin 7-*O*-glucoside
**8**	5.04	C_25_H_24_O_12_	517.1343	[M + H]^+^	515.1198	[M − H]^−^	3,4-dicaffeoylquinic acid
**9**	5.22	C_20_H_18_O_11_	435.0919	[M + H]^+^	433.0778	[M − H]^−^	quaijaverin
**10**	5.30	C_25_H_24_O_12_	517.1342	[M + H]^+^	515.1199	[M − H]^−^	3,5-dicaffeoylquinic acid
**11**	5.45	C_24_H_22_O_14_	535.1084 449.1077	[M + H]^+^ [M − Mal + H]^+^	533.0942	[M − H]^−^	luteolin 7-*O*-(6″-malonylglucoside)
**12**	5.49	C_25_H_24_O_12_	517.1339	[M + H]^+^	515.1196	[M − H]^−^	4,5-dicaffeoylquinic acid
**13**	5.94	C_14_H_14_O_5_	263.0912	[M + H]^+^	-	-	khellactone
**14**	6.92	C_21_H_22_O_6_	371.1488	[M + H]^+^	369.1344	[M − H]^−^	xanthoangelol E
**15**	8.06	C_12_H_8_O_4_	217.0496	[M + H]^+^	-	-	bergapten or methoxsalen
**16**	8.85	C_21_H_22_O_5_	355.1538	[M + H]^+^	353.1396	[M − H]^−^	xanthoangelol D
**17**	9.98	C_14_H_14_O_3_	231.1015	[M + H]^+^	229.0871	[M − H]^−^	osthenol
**18**	10.23	C_19_H_20_O_6_	362.1595	[M + NH_4_]^+^	-	-	(+)-laserpitin or (−)-isolaserpitin
**19**	10.59	C_19_H_20_O_6_	362.1595	[M + NH_4_]^+^	-	-	daucoidin A ^b^
**20**	10.78	C_19_H_20_O_6_	362.1595	[M + NH_4_]^+^	-	-	daucoidin B ^b^

^a,b^ Interchangeable. ^c^ Comparison with a standard sample.

## Data Availability

The data presented in this study and the samples of the compounds are available upon request from the corresponding author.

## Supplementary Materials

The following supporting information can be downloaded at: https://www.mdpi.com/article/10.3390/molecules30061394/s1, Table S1: MS/MS data of compounds in extract of *A. keiskei* leaves. Table S2: MS/MS data of products in the extract of *A. keiskei* leaves after incubation with methylglyoxal. Figures S1–S7 Possible fragmentation pathways of compounds (positive ion mode).
