# Supplementary material for: Anti-Glycation Activities of Angelica keiskei Leaves
Source: Molecules. 2025 Mar 20;30(6):1394. doi: 10.3390/molecules30061394 (PMC11945142; doi:10.3390/molecules30061394)
Supplement: Supplementary file 1 [file molecules-30-01394-s001.zip › molecules-3480380-supplementary.pdf]

Supplementary Material

Anti-glycation Activities of *Angelica keiskei* Leaves

Yuno Takemoto, Takashi Kikuchi*, Wenjun Qi, Mi Zhang, Kouharu Otsuki, and Wei Li *

Faculty of Pharmaceutical Sciences, Toho University, Miyama 2-2-1, Funabashi, Chiba 274-8510, Japan
* Correspondence: liwei@phar.toho-u.ac.jp (W. Li), takashi.kikuchi@phar.toho-u.ac.jp (T. Kikuchi)

Content

・Table S1 MS/MS data of compounds in extract of *Angelica keiskei* leaves
・Table S2 MS/MS data of products in the extract of *Angelica keiskei* leaves after incubation with methylglyoxal.
・Figure S1 Possible fragmentation pathways of **1**, **2**, **8**, **10**, and **12** (positive ion mode).
・Figure S2 Possible fragmentation pathways of **3** and **4** (positive ion mode).
・Figure S3 Possible fragmentation pathways of **13** and **18** (positive ion mode).
・Figure S4 Possible fragmentation pathway of **14** (positive ion mode).
・Figure S5 Possible fragmentation pathway of **16** (positive ion mode).
・Figure S6 Possible fragmentation pathway of **17** (positive ion mode).
・Figure S7 Possible fragmentation pathways of **19** and **20** (positive ion mode).

Table S1. MS/MS data of compounds in extract of *Angelica keiskei* leaves.

No.	MS/MS (Positive, HCD 40 eV)	MS/MS (Positive, HCD 60 eV)	Identification
**1**	163 (100), 145 (10), 135 (6), 117 (2), 89 (1)	193 (9), 175 (9), 163 (99), 145 (46), 137 (2), 135 (100), 133 (7), 119 (1), 117 (41), 111 (1), 107 (11), 95 (1), 89 (30), 83 (2), 79 (6), 63 (1)	chlorogenic acid
**2**	177 (100), 149 (3), 145 (58), 117 (5), 89 (2)	177 (25), 163 (2), 149 (25), 145 (100), 135 (5), 117 (62), 103 (1), 91 (1), 89 (16), 83 (2), 67 (1), 63 (1)	feruloylquinic acid
**3**	229 (100), 213 (2), 201 (2), 183 (1)	229 (100), 227 (5), 215 (10), 213 (62), 211 (27), 201 (10), 199 (4), 197 (5), 189 (3), 185 (8), 183 (10), 175 (2), 173 (10), 171 (2), 169 (5), 167 (3), 165 (4), 159 (4), 155 (18), 153 (2), 147 (3), 145 (8), 143 (2), 141 (3), 131 (11), 129 (4), 117 (3), 115 (3), 105 (4), 71 (2), 69 (3)	nodakenin[a]
**4**	247 (15), 229 (100), 187 (7), 175 (3), 153 (32), 135 (22), 127 (20), 93 (14), 85 (21), 71 (15), 57 (2)	247 (5), 229 (100), 213 (81), 211 (32), 185 (17), 175 (23), 155 (22), 131 (14), 105 (7), 85 (13), 69 (9), 55 (4), 53 (2)	marmesinin[a]
**5**	287 (100), 85 (6), 71 (5)	287 (100), 241 (1), 171 (2), 161 (1), 153 (9), 137 (1), 135 (3), 85 (5), 71 (5), 57 (1)	luteolin 7-*O*-rutinoside
**6**	303 (100), 229 (2), 165 (1), 127 (3), 109 (1), 99 (1), 97 (5), 91 (1), 85 (16), 81 (2), 73 (1), 69 (4), 61 (3)	303 (100), 285 (12), 257 (20), 229 (42), 219 (3), 201 (20), 165 (22), 153 (47), 137 (35), 111 (11), 97 (20), 85 (53), 69 (31), 57 (7), 53 (6)	quercetin 3-*O*-glucoside
**7**	287 (100)	287 (100), 269 (1), 241 (2), 171 (2), 161 (2), 153 (17), 137 (1), 135 (7), 69 (1), 67 (1)	luteolin 7-*O*-glucoside
**8**	163 (100), 145 (10), 135 (9), 117 (1)	163 (81), 145 (38), 135 (100), 123 (2), 117 (38), 89 (36), 63 (2), 53 (1)	3,4-dicaffeoyl quinic acid
**9**	303 (100), 257 (1), 115 (1), 73 (26), 61 (3)	303 (100), 285 (10), 257 (22), 247 (5), 229 (38), 219 (3), 201 (15), 183 (9), 173 (3), 165 (16), 163 (6), 153 (38), 149 (4), 137 (31), 121 (9), 111 (8), 109 (3), 95 (3), 85 (3), 73 (76), 69 (4), 61 (10), 57 (7)	quaijaverin
**10**	163 (100), 145 (9), 135 (2), 117 (2)	163 (97), 145 (45), 135 (100), 117 (39), 107 (9), 95 (2), 89 (31), 79 (5), 63(1)	3,5-dicaffeoyl quinic acid
**11**	315 (3), 287 (100), 163 (7)	315 (2), 287 (100), 269 (1), 241 (2), 179 (1), 171 (4), 163 (5), 161 (2), 153 (13), 145 (2), 137 (2),	luteolin 7-*O*-(6″-

		135 (10), 117 (2), 107 (1), 93 (1), 91 (1), 89 (2), 85 (1), 81 (1), 79 (1), 69 (1), 67 (1)	malonylglucoside)
**12**	163 (100), 145 (11), 135 (9), 117 (2), 89 (1)	163 (89), 145 (41), 135 (100), 117 (40), 95 (2), 89 (35), 63 (1)	4,5-dicaffeoylquinic acid
**13**	245 (4), 227 (1), 215 (2), 203 (100), 191 (8), 187 (11), 175 (10), 59 (14)	203 (72), 191 (22), 175 (100), 159 (5), 147 (14), 135 (10), 107 (25), 91 (6), 59(31)	khellactone
**14**	311 (15), 295 (17), 233 (8), 205 (100), 195 (7), 191 (9), 181 (11), 179 (63), 175 (14), 163 (13), 147 (95), 133 (9), 119 (5)	205 (29), 197 (5), 195 (33), 191 (11), 181 (39), 179 (56), 177 (8), 175 (16), 163 (42), 147 (3), 147 (100), 133 (16), 133 (32), 119 (70), 107 (4), 105 (14), 91 (22), 59 (8)	xanthoangelol E
**15**	217 (100), 203 (8), 202 (51), 189 (8), 185 (8), 174 (5), 173 (5), 161 (14), 158 (1), 145 (1), 117 (2), 115 (2)	217 (14), 203 (18), 189 (7), 185 (7), 175 (11), 173 (3), 161 (49), 157 (4), 147 (1), 145 (1), 145 (2), 133 (2), 131 (6), 129 (1), 117 (2), 115 (10), 105 (3), 91 (2), 89 (1)	bergapten or methoxsalen
**16**	337 (3), 295 (4), 283 (7), 235 (38), 223 (2), 217 (33), 191 (3), 185 (1), 181 (70), 175 (1), 163 (100), 151 (1), 147 (9), 133 (7), 105 (2)	235 (4), 217 (16), 181 (78), 163 (100), 147 (11), 133 (99), 119 (8), 105 (56), 91 (3)	xanthoangelol D
**17**	231 (48), 189 (2), 175 (100), 147 (2), 103 (1), 69 (1)	231 (4), 189 (2), 187 (3), 177 (2), 175 (100), 147 (17), 133 (2), 133 (2), 131 (1), 119 (18), 107 (6), 105 (1), 103 (16), 91 (11), 79 (4), 69(9)	osthenol
**18**	245 (4), 203 (100), 187 (10), 175 (4), 59 (7)	203 (100), 187 (11), 175 (90), 159 (2), 147 (7), 143 (1), 131 (6), 119 (1), 115 (1), 91 (3), 59 (17)	(+)-laserpitin or (−)-isolaserpitin
**19**	245 (2), 227 (23), 215 (3), 203 (3), 191 (4), 189 (2), 187 (1), 175 (2), 83 (100), 55 (38)	227 (21), 199 (14), 187 (21), 175 (22), 159 (10), 143 (7), 131 (16), 107 (11), 83 (43), 59 (7), 55 (100), 53 (2)	daucoidin A[c]
**20**	245 (6), 227 (66), 217 (1), 215 (2), 203 (6), 199 (4), 191 (8), 189 (4), 187 (27), 175 (6), 159 (1), 131 (1), 101 (3), 83 (100), 59 (3), 55 (44), 53(1)	227 (21), 199 (14), 187 (21), 175 (22), 159 (10), 143 (7), 131 (16), 107 (11), 83 (43), 59 (7), 55 (100), 53 (2)	daucoidin B[c]

Table S2. MS/MS data of products in the extract of *Angelica keiskei* leaves after incubation with methylglyoxal.

t_R min	Precursor ion *m/z*	MS/MS (Positive, HCD 40 eV)	MS/MS (Positive, HCD 60 eV)
3.58	609[a]	411 (12), 383 (82), 365 (1), 355 (100), 341 (1), 337 (2), 137 (5), 127 (1), 97 (4), 91 (5), 85 (7), 73 (3), 61 (8)	383 (4), 355 (100), 355 (3), 337 (5), 327 (3), 313 (3), 309 (3), 299 (7), 287 (5), 281 (3), 233 (4), 205 (24), 189 (6), 163 (4), 138 (5), 137 (59), 97 (8), 91 (4), 85 (12), 73 (6), 69 (9), 61 (26)
3.66	609[a]		383 (4), 355 (100), 339 (3), 337 (4), 327 (4), 313 (4), 309 (3), 299 (7), 287 (7), 281 (4), 259 (4), 233 (4), 205 (29), 189 (6), 187 (4), 163 (5), 137 (58), 97 (9), 85 (13), 73 (10), 69 (9), 61 (24)
3.74	609[a]		355 (100), 337 (5), 327 (5), 313 (3), 299 (6), 287 (5), 259 (4), 233 (4), 205 (25), 189 (6), 187 (3), 163 (5), 138 (4), 137 (50), 97 (8), 85 (18), 81 (6), 69 (14), 61 (14), 57 (4), 53 (3)
4.05	607[b]	427 (3), 399 (2), 385 (13), 384 (10), 357 (100), 353 (1), 341 (10), 339 (1), 329 (68), 311 (3), 303 (2), 301 (2), 137 (4), 127 (1), 97 (6), 91 (7), 85 (11), 73 (4), 69 (2), 61 (13)	357 (10), 329 (100), 273 (10), 179 (28), 137 (61), 61 (43)
4.11	607[b]		
4.32	607	427 (3), 399 (10), 385 (8), 381 (2), 357 (23), 353 (1), 329 (100)	329 (100), 311 (6), 179 (6), 137 (17), 61 (19)
4.54	605	397 (2), 383 (5), 373 (25), 369 (2), 355 (21), 327 (7), 303 (100), 127 (1), 97 (5), 91 (6), 85 (12), 73 (4), 69 (2), 61 (12)	355 (3), 327 (4), 303 (100), 285 (5), 281 (3), 257 (9), 229 (9), 205 (5), 201 (3), 165 (6), 153 (6), 137 (16), 121 (3), 97 (9), 85 (15), 73 (6), 69 (7), 61 (27), 57 (3)
4.44	667	359 (3), 341 (65), 313 (100), 295 (1), 287 (3), 207 (6), 137 (1), 85 (6), 71 (6), 57 (1)	341 (2), 313 (100), 295 (2), 287 (2), 207 (7), 197 (1), 179 (12), 161 (1), 137 (5), 135 (2), 93 (3), 85 (4), 71 (5), 69 (2)

4.62	521[b]	341 (24), 313 (100), 287 (3)	313 (100), 287 (3), 179 (9), 137 (5), 69 (3)
4.67	521[b]		
4.77	521[b]		

[a]The average value due to the proximity of the peaks at 40 eV.

[b]The average value due to the proximity of the peaks at 40 and 60 eV

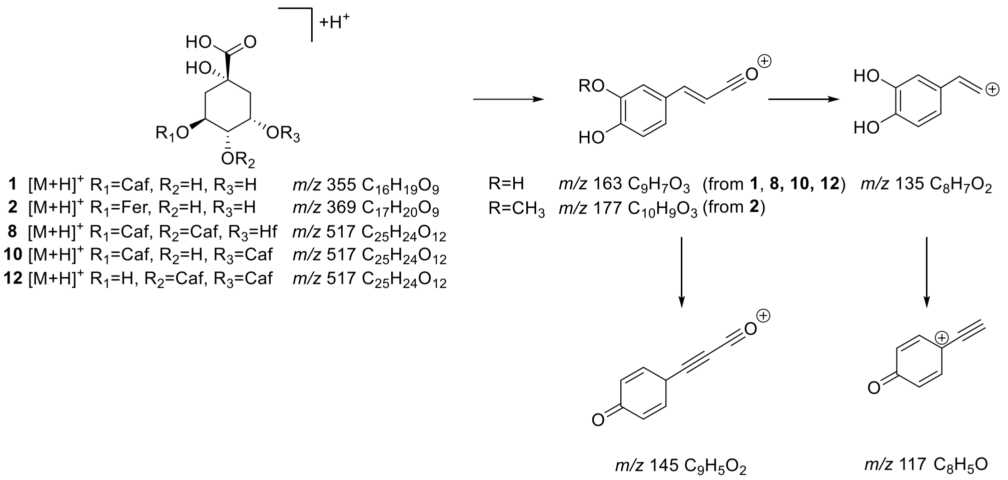

Figure S1. Possible fragmentation pathways of **1, 2, 8,10**, and **12** (positive ion mode).

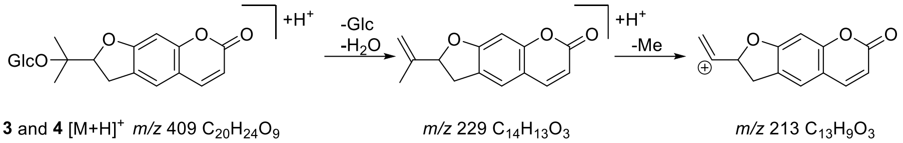

Figure S2. Possible fragmentation pathways of **3** and **4** (positive ion mode).

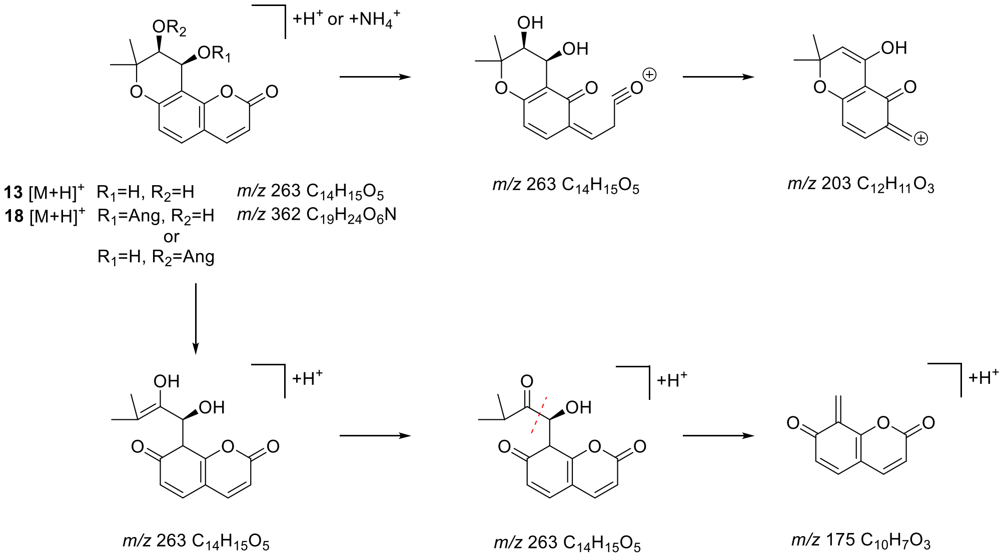

Figure S3. Possible fragmentation pathways of **13** and **18** (positive ion mode).

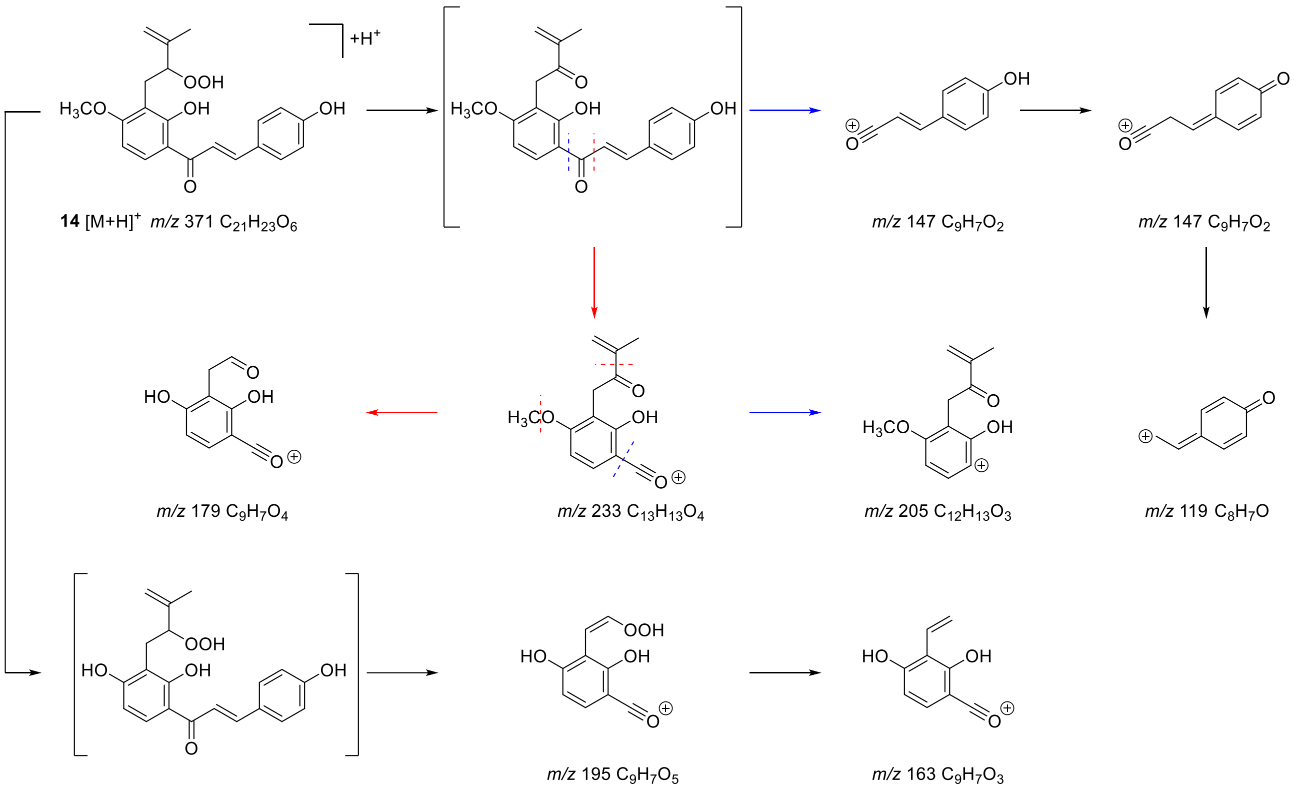

Figure S4. Possible fragmentation pathways of **14** (positive ion mode).

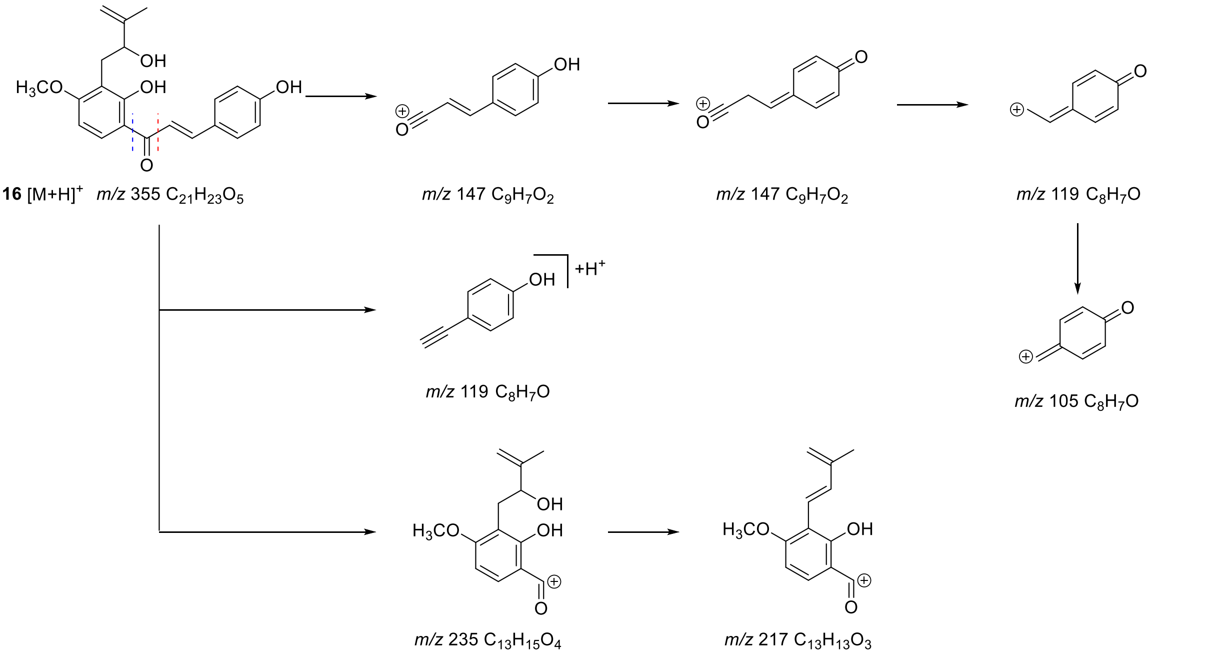

Figure S5. Possible fragmentation pathways of **16** (positive ion mode).

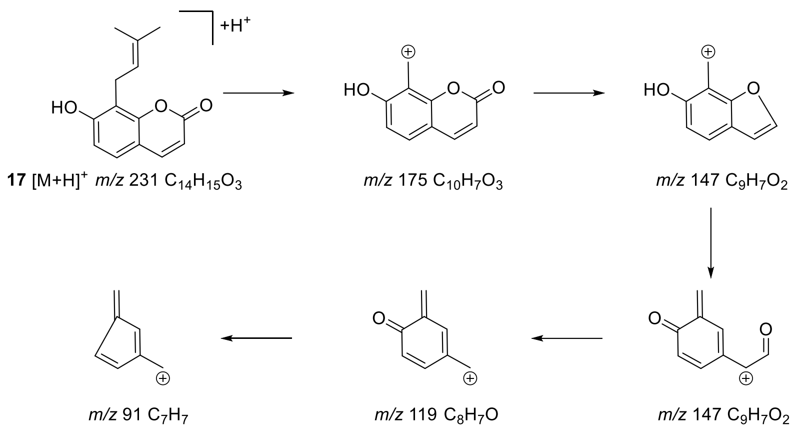

Figure S6. Possible fragmentation pathways of **17** (positive ion mode).

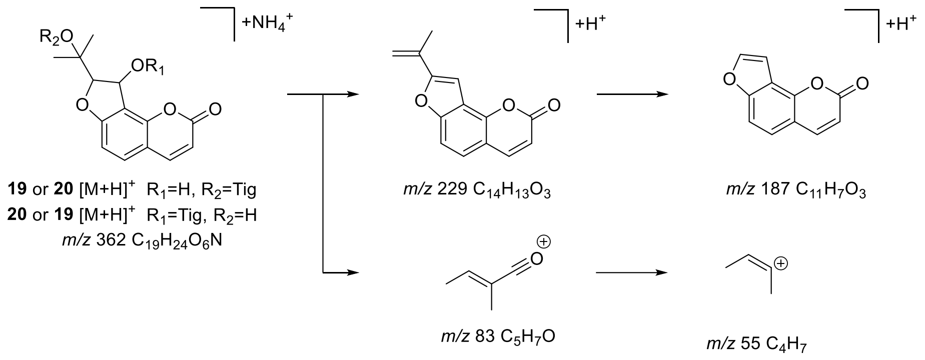

Figure S7. Possible fragmentation pathways of **19** and **20** (positive ion mode).
